# Transcriptomic analyses of ovarian clear-cell carcinoma with concurrent endometriosis

**DOI:** 10.3389/fendo.2023.1162786

**Published:** 2023-08-09

**Authors:** Kaitlyn E. Collins, Xiyin Wang, Yuliya Klymenko, Noah B. Davis, Maria C. Martinez, Chi Zhang, Kaman So, Aaron Buechlein, Douglas B. Rusch, Chad J. Creighton, Shannon M. Hawkins

**Affiliations:** ^1^ Department of Biochemistry and Molecular Biology, Indiana University School of Medicine, Indianapolis, IN, United States; ^2^ Indiana University Melvin and Bren Simon Comprehensive Cancer Center, Indiana University School of Medicine, Indianapolis, IN, United States; ^3^ Department of Obstetrics and Gynecology, Indiana University School of Medicine, Indianapolis, IN, United States; ^4^ Mayo Clinic Graduate School of Biomedical Sciences, Mayo Clinic College of Medicine and Science, Rochester, MN, United States; ^5^ Department of Anatomy, Cell Biology, and Physiology, Indiana University School of Medicine, Indianapolis, IN, United States; ^6^ Department of Medical and Molecular Genetics, Indiana University School of Medicine, Indianapolis, IN, United States; ^7^ Center for Computational Biology and Bioinformatics, Indiana University School of Medicine, Indianapolis, IN, United States; ^8^ Center for Genomics and Bioinformatics, Indiana University, Bloomington, IN, United States; ^9^ Department of Medicine, Baylor College of Medicine, Houston, TX, United States

**Keywords:** endometriosis, ovarian endometrioma, ovarian clear-cell carcinoma, transcriptomic profiling, miRNA

## Abstract

**Introduction:**

Endometriosis, a benign inflammatory disease whereby endometrial-like tissue grows outside the uterus, is a risk factor for endometriosis-associated ovarian cancers. In particular, ovarian endometriomas, cystic lesions of deeply invasive endometriosis, are considered the precursor lesion for ovarian clear-cell carcinoma (OCCC).

**Methods:**

To explore this transcriptomic landscape, OCCC from women with pathology-proven concurrent endometriosis (*n* = 4) were compared to benign endometriomas (*n* = 4) by bulk RNA and small-RNA sequencing.

**Results:**

Analysis of protein-coding genes identified 2449 upregulated and 3131 downregulated protein-coding genes (DESeq2, *P*< 0.05, log2 fold-change > |1|) in OCCC with concurrent endometriosis compared to endometriomas. Gene set enrichment analysis showed upregulation of pathways involved in cell cycle regulation and DNA replication and downregulation of pathways involved in cytokine receptor signaling and matrisome. Comparison of pathway activation scores between the clinical samples and publicly-available datasets for OCCC cell lines revealed significant molecular similarities between OCCC with concurrent endometriosis and OVTOKO, OVISE, RMG1, OVMANA, TOV21G, IGROV1, and JHOC5 cell lines. Analysis of miRNAs revealed 64 upregulated and 61 downregulated mature miRNA molecules (DESeq2, *P*< 0.05, log2 fold-change > |1|). MiR-10a-5p represented over 21% of the miRNA molecules in OCCC with endometriosis and was significantly upregulated (NGS: log2fold change = 4.37, *P* = 2.43e-18; QPCR: 8.1-fold change, *P*< 0.05). Correlation between miR-10a expression level in OCCC cell lines and IC50 (50% inhibitory concentration) of carboplatin *in vitro* revealed a positive correlation (R^2 = ^0.93). MiR-10a overexpression *in vitro* resulted in a significant decrease in proliferation (*n* = 6; *P<* 0.05) compared to transfection with a non-targeting control miRNA. Similarly, the cell-cycle analysis revealed a significant shift in cells from S and G_2_ to G_1_ (*n* = 6; *P<* 0.0001). Bioinformatic analysis predicted that miR-10a-5p target genes that were downregulated in OCCC with endometriosis were involved in receptor signaling pathways, proliferation, and cell cycle progression. MiR-10a overexpression *in vitro* was correlated with decreased expression of predicted miR-10a target genes critical for proliferation, cell-cycle regulation, and cell survival including [*SERPINE1* (3-fold downregulated; *P<* 0.05), *CDK6* (2.4-fold downregulated; *P<* 0.05), and *RAP2A* (2-3-fold downregulated; *P<* 0.05)].

**Discussion:**

These studies in OCCC suggest that miR-10a-5p is an impactful, potentially oncogenic molecule, which warrants further studies.

## Introduction

Previous studies have shown that each histotype of epithelial ovarian carcinoma, including high-grade serous, endometrioid, and clear-cell carcinomas, are transcriptomically distinct (1, 2). Large-scale molecular analyses of high-grade serous ovarian carcinomas showed unique classifications of tumors based on integrating multi-platform profiling (3). Molecular profiling of the endometriosis-associated ovarian carcinomas, including ovarian endometrioid and clear-cell carcinomas, showed frequent loss-of-function mutations in *ARID1A* (4–6). Previous work showed a unique transcriptomic profile in endometrioid ovarian carcinoma from women with concurrent endometriosis compared to those without concurrent endometriosis, supporting the role of the endometriotic microenvironment in ovarian cancer development (7, 8).

Endometriosis, a benign, chronic inflammatory condition where endometrial-like tissue grows outside the uterus, is a significant and potentially modifiable risk factor for ovarian cancer development (9, 10). Women with any amount or anatomical location of endometriosis have an increased risk of developing ovarian endometrioid (3.1-fold) or clear-cell (5.1-fold) carcinoma (11, 12). Specifically, women with ovarian endometriomas or deep infiltrating endometriotic lesions of the ovary are at even higher risk for developing ovarian endometrioid (4.7-fold) or clear-cell (10.1-fold) carcinoma (11). OCCC is a rare histological subtype composing 5-25% of ovarian malignancies, with the wide variation thought to be due to the subjective evaluation of histologic features and country-specific differences in prevalence (13–15).

Unlike the large sample size high-grade serous transcriptomic profiling studies focusing only on high-grade serous histology, transcriptomic profiling of OCCC is more limited in sample size or primarily used to show differences between transcriptomic profiles of different histological types of epithelial ovarian cancers (3, 16–21). Significantly, up to 50% of OCCCs are associated with endometriosis (22, 23). However, most transcriptomic studies of primary tumors classified as OCCC do not characterize samples as coming from women with concurrent endometriosis, pathology-proven endometriosis, or even a history of endometriosis. Only recently was a large sample size, OCCC-focused, multi-platform study performed that characterized samples as from women with a history of endometriosis (16). In this study, samples from women with a history of endometriosis were more likely to have loss-of-function mutations in *ARID1A.* In contrast, those with p53 mutations were likely to have poorer outcomes (16). While the tumors transcriptomically clustered into two groups – a traditional OCCC group and an aggressive p53-mutant high-grade serous-like group (16), the study did not discern particular transcriptomic contributions in samples from women with a history of endometriosis. To fill this gap, we focused our transcriptomic profiles on OCCC with pathology-proven concurrent endometriosis using both bulk RNA and miRNA sequencing.

MicroRNAs (miRNAs) are single-stranded, 22-24 nucleotide RNA molecules that function through an eight nucleotide seed sequence to modulate gene networks (24). MiRNAs are dysregulated in malignant and benign gynecological diseases and play impactful, functional roles in endometriomas and OCCC cell lines (24–27). For example, previous work has shown that endometriomas have distinct miRNA profiles and specific miRNAs, including miR-29c, play critical roles in uterine dysfunction (26). As another example, next-generation sequencing of OCCC cell lines showed that miR-100 played a critical role in rapamycin resistance *in vitro* (27). These limited studies suggest that miRNA molecules play essential roles. However, miRNA profiles in OCCC with concurrent endometriosis have not been examined. As a multi-platform approach, we integrated small RNA sequencing for miRNAs on matched samples of OCCC from women with pathology-confirmed concurrent endometriosis. From our list of dysregulated miRNAs, miR-10a was chosen to explore potential cellular and molecular associations in OCCC cell lines.

## Materials and methods

### Institutional review board approval for collection of human tissues and metadata

The expedited protocol to obtain and use tissues for this study was reviewed and approved by the Institutional Review Board (IRB) at Indiana University (#1812764043). The participants provided written informed consent to participate in this study. De-identified flash-frozen specimens, surgical pathology reports, and demographic data were obtained from tissue banks or previous studies (26). Tissue banks included the NRG Oncology Biospecimen Bank (NRG BB) and the Biospecimen Collection and Banking Core (BC^2^) at the Indiana University Melvin and Bren Simon Comprehensive Cancer Center (IUSCCC). **Supplementary Table S1** lists the metadata and associated experiments for each de-identified human tissue sample.

OCCC with concurrent endometriosis and OCCC without endometriosis were pure clear-cell histology samples collected from adnexal masses. Tumors with mixed histology (*i.e.*, clear-cell with endometrioid or clear-cell with serous) were not included in these studies. Inclusion criteria for OCCC with concurrent endometriosis samples were defined as the explicit mention of endometriosis on the surgical pathology report. Per banking protocols, ovarian cancer samples were taken away from obvious pathologies such as necrotic tissue or endometriosis. The malignant samples were 50-90% malignant cells (**Supplementary Table S1**). Endometrioma cyst wall tissues were collected as described previously (26).

### Next-generation sequencing studies

Total RNA was isolated from 50-100 mg of fresh frozen tissue using the *mir*Vana miRNA Isolation Kit with phenol (Thermo Fisher Scientific, Waltham, MA). RNA was treated with the Turbo DNA-*free* Kit (Thermo Fisher Scientific). RNA quality control was assessed using a 2100 Bioanalyzer (Agilent Technologies, Palo Alto, CA) at the Center for Medical Genomics at Indiana University School of Medicine (Indianapolis, IN). High-quality RNA samples were sent to the Center for Genomics and Bioinformatics at Indiana University (Bloomington, IN). Poly-A RNA libraries were constructed using mRNA Stranded TruSeq protocol (Illumina, San Diego, CA). Small RNA library construction was performed using the TruSeq Small RNA kit (Illumina). Purified libraries were visualized and quantified using a TapeStation HSD1000 (Agilent Technologies).

For poly-A bulk RNA sequencing analysis, NextSeq reads were trimmed using fastp (version 0.23.2) with parameters “-l 17 -g -p” (28). The resulting reads were mapped against GRCh38 using HISAT2 version 2.2.1 with default parameters (29). HISAT uses Bowtie2, based on the Burrows-Wheeler transform algorithm, for sequence alignment and allows for mapping across exon junctions (30). Read counts for each gene were created using featureCounts from the Subread package version 2.0.3 with the parameters “-O -M –primary –largestOverlap -s 2” and Gencode v42 as the annotation (31–33). For small RNA sequencing analysis, NextSeq reads were trimmed using fastp (version 0.23.2) with parameters “-l 17 -g -p” (28). MiRDeep2 version 2.0.0.8 was used to map the resulting reads against GRCh38 and miRBase version 22 as a reference to detect known, mature miRNA sequences (34–36). MiRDeep2 uses Bowtie to perform mapping of the reads and includes tools for the identification and quantification of miRNAs (37). Bowtie version 1.3.0 was the version of Bowtie installed. Differential expression analysis for bulk RNA and miRNA was performed using the DESeq2 package (version 1.36.0) in R/Bioconductor (R version 4.2.0) (38). Transcriptomic data have been deposited into the Gene Expression Omnibus (GSE230956). Figures from poly-A bulk RNA and small RNA sequencing analysis were created using R (version 4.2.0) and R libraries: ggplot2, complex heatmap, and ggrepel.

### Quantitative PCR for mRNA and miRNA expression

Total RNA was extracted from 50-100 mg of fresh frozen tissue or cultured cells using the miRNeasy Mini Kit (Qiagen, Hilden Germany) following the manufacturer’s protocol with on-column RNase-Free DNase Set (Qiagen) or previously extracted DNase-treated RNA using the *mir*Vana kit described above. A NanoDrop ND-1000 (Thermo Fisher Scientific) was used for the determination of RNA quantity and purity. For mRNA expression experiments, 1000 ng of DNase-treated RNA was reverse transcribed in a 20 µL reaction using 50 units MultiScribe Reverse Transcriptase (Thermo Fisher Scientific), 1X reverse transcriptase Buffer (Thermo Fisher Scientific), 0.5 mM deoxynucleotide triphosphate (Thermo Fisher Scientific), 6 units RNase Inhibitor (Thermo Fisher Scientific), and 2.5 µM random hexamers (Thermo Fisher Scientific) on a 2720 Thermo Cycler (Thermo Fisher Scientific): 10 minutes at 25°C, 30 minutes at 48°C, and 5 minutes at 95°C. Samples were diluted 1:5 for qPCR. QPCR was performed using 2 μL of diluted cDNA using either SYBR Green PCR Master Mix (Thermo Fisher Scientific) with previously published primers (39) or custom-designed primers (**Supplementary Table S2**) in a reaction volume of 10 μl. Only custom-designed primer pairs specific for the gene of interest, intron-spanning, with a primer efficiency of 80-110%, lacking primer-dimers, and R^2^ >0.95 were used (40, 41). mRNA experiments were normalized to the human β-actin (*ACTB*) (39). For miRNA expression experiments, total RNA (25 ng) was reverse transcribed using the TaqMan MicroRNA Reverse Transcription Kit (Thermo Fisher Scientific) in a reaction volume of 15 μl. Mature miRNA expression was performed using TaqMan mature microRNA assays on undiluted cDNA. U6 snRNA was used for normalization (26, 42). **Supplementary Table S2** lists the TaqMan assays used.

Both mRNA and miRNA assays were run on a QuantStudio 3 Real-Time PCR Instrument (Thermo Fisher Scientific) with reaction conditions as follows: 2 minutes at 50°C, 10 minutes at 95°C, followed by 40 cycles of 15 seconds at 95°C (denaturation), and 1 minute at 60°C (annealing/extension). All SYBR Green assays ran dissociation curves to detect primer dimers. Each sample was analyzed in duplicate. Expression fold change calculations utilized the 2^-ΔΔCT^ method (43). Data were plotted as mean ± SEM, and statistical analyses were performed with GraphPad Prism (Dotmatics, Boston, MA). *P*< 0.05 was considered statistically significant. Power analyses were performed using G*Power (version 3.1.9.7) (44, 45). *Post-hoc* analysis of A2780 and A2780CR5 miR-10a expression, with a type I error set at 0.05, found that we had greater than 95% power to detect a three-fold change with effect size d= 9.6 with a sample size of two in each group using a two-tailed t-test. For tissue miR-10a expression, due to greater variability, a total sample size of 24 was calculated to achieve greater than 80% power to detect a 0.7 effect size f and a type I error set at 0.05 using a one-way ANOVA.

### Tissue-cell line transcriptomics data comparison analyses

To compare tissue and cell line collected from different studies, we computed the pathway activity scores (PAS) of an extensive collection of canonical biological pathways for each sample and utilized the PAS to assess the similarity between samples. We assume that the cell lines and tissue samples of high similarity should have a similar profile in more similarly activated cellular pathways. Noting that cell line samples do not have the biological characteristics of tumor microenvironments, we excluded stromal genes and related pathways from the PAS analysis. Specifically, canonical gene sets were downloaded from MSigDB version 6 c2, containing 1329 gene sets (46–48). Cancer Cell Line Encyclopedia (CCLE) cell line RNA-seq gene expression data were downloaded from the Broad Institute (49). The housekeeping genes and immune and stromal cell marker genes derived from our previous analysis were excluded (50, 51). Pathway activity scores (PAS) were assigned using the following function for each sample and pathway. For a given gene expression profile *x* ^1×^ *^N^* of *N* genes and a pathway *P* as a set of genes, denote *y* ^1×^ *^N^* as the sorted *x* ^1×^ *^N^* in the decreasing order and *i_g_* as the rank of gene *g* in *y*, the pathway activity score of *P* on *x*, denoted as *PAS*(*x,P,K*) is computed by


$$PAS(x,P,K)=\frac{\sum_{g\in P}\max\{\frac{K-i_{g}}{|P|},0\}}{K}$$

where *K* is the hyperparameter in this study. Here, K is set as 3000. Here, the *PAS* can be viewed as a normalized and weighted sum of the rank of the pathway genes whose expression is within the top K=3000 rank. PAS is computed for each pathway and each sample. Then Pearson Correlation Coefficients of the PAS of all pathways were computed between samples and used as their molecular similarity measure.

### Ovarian cancer cell lines

ES-2 (52, 53), TOV-21G (54), and IGROV-1 (55) were obtained from the American Type Culture Collection (ATCC, Manassas, VA). SKOV3ip1 (56) was obtained from the Cytogenetics and Cell Authentication Core at the University of Texas M.D. Anderson Cancer Center (Houston, TX, USA). OVISE (57), OVAS (58), and OVTOKO (57) were generously obtained from Dr. Hiroshi Minaguchi (Yokohama City University, Yokohama, Japan). KK (59) was generously obtained from Dr. Yoshihiro Kikuchi (National Defense Medical College, Tokorozawa, Japan). SMOV-2 (60) was generously obtained from Dr. Tomohiro Iida (St. Marianna University, Kawasaki, Japan). A2780 (61) and A2780CR5 (62) were provided by Dr. Kenneth P. Nephew (Indiana University, Bloomington, IN, USA). SKOV3 (63) was generously obtained from Dr. Salvatore Condello (Indiana University School of Medicine, Indianapolis, IN, USA). RMG-I (64) was generously obtained from Dr. Samuel C. Mok (The University of Texas MD Anderson Cancer Center, Houston, TX, USA). Cell line authentication was confirmed using a short tandem repeat (STR) marker profile (IDEXX BioAnalytics, Westbrook, ME) within six months of experiments and tested for mycoplasma contamination monthly (MycoAlert Plus Mycoplasma Detection Kit, Lonza, Switzerland).

KK, OVISE, OVTOKO, IGROV-1, RMG-I, A2780, A2780CR5, and SKOV3 were maintained in RPMI 1640 (Thermo Fisher Scientific). OVAS was maintained in DMEM/F12 (Thermo Fisher Scientific). TOV-21G was maintained in a 1:1 ratio of Medium 199 to MCBD 105 (Sigma-Aldrich, St. Louis, MO). ES-2 was grown in McCoy’s (Thermo Fisher Scientific). All cell lines were supplemented with 1% penicillin and streptomycin (Thermo Fisher Scientific) and 10% fetal bovine serum (Atlanta Biologicals, Minneapolis, MN) except for TOV-21G, which was supplemented with 15%. All cells were cultured in a humidified incubator at 37°C with 5% carbon dioxide. **Supplementary Table S3** lists the published common genetic mutations, drug responses, and the experimental uses for each of the cell lines used in this manuscript.

### Carboplatin cytotoxicity assays

Carboplatin cytotoxicity assays were performed using the CellTiter 96 AQueous One Solution Cell Proliferation Assay (MTS) (Promega, Madison, WI). Cells were plated 1x10^3^ cells per 96-well. After 24 hours, cells were treated with ten increasing (5-200 µM) doses of carboplatin [cis-diammine (1,1-cyclobutane-dicarboxylate) platinum, (C2358, Sigma)] diluted in tissue culture grade water (Thermo Fisher Scientific) in triplicate. Following 72-hours of carboplatin treatment, absorbance was read on a Synergy H1 Hybrid Reader (BioTek, Winooski, VT), background absorbance was subtracted, and data were presented as normalized to vehicle control. GraphPad Prism version 9.3.0 (Dotmatics) was used to calculate an IC50 (50% inhibitory concentration). GraphPad Prism (Dotmatics) was used to calculate the correlation between IC50 and miR-10a-5p expression. With a type I error set at 0.05, we will have 90% power to detect a correlation of 0.85 with a total sample size of 8. Figures were created using GraphPad Prism (Dotmatics).

### miRNA target prediction

Putative miRNA:mRNA pairs were facilitated using SigTerms (65) with input from TargetScan (66–68), miRDB (69, 70), and miRTarBase (71). Putative target genes were further curated for potential as impactful miR-10a-5p targets using hand annotation.

### MiR-10a-5p mimic transfection of human OCCC cell lines

Each cell line underwent optimization of transfection conditions using the siGLO Red Transfection Indicator (Horizon Discovery, Cambridge, United Kingdom) to determine the optimum amount of lipid transfection reagent, miRNA mimic concentration, and initial cell density. Cells were seeded at a density of 2-3x10^5^ cells per well of a 6-well plate. After 24 hours, cells were transfected using Lipofectamine RNAiMAX Transfection Reagent (Thermo Fisher Scientific) with 100 nM hsa-miR-10a-5p mimic (*mir*Vana miRNA mimic, Assay ID MC10787) or 100 nM negative control (*mir*Vana miRNA mimic, Negative Control #1, catalog #4464058).

Transfected cells were used simultaneously for four different endpoints: confirmation of miR-10a overexpression, cellular proliferation, cell cycle analysis, and associated putative target gene expression by qPCR. To confirm miR-10a overexpression and associated putative target gene expression, cells were lysed at 24 hours after transfection for RNA isolation. To evaluate the effects of miR-10a overexpression on proliferation, 24 hours after transfection, cells were seeded into a 96-well plate at a density of 1000 cells/well. Cellular proliferation was measured using CellTiter 96 AQueous One Solution Cell Proliferation Assay (MTS) (Promega) in triplicate at 24-hour intervals. Absorbance was read with the Synergy H1 Hybrid Reader (BioTek). Proliferation was plotted as the percent of viable cells as a function of time using GraphPad Prism (Dotmatics). To assess the effects of miR-10a overexpression on the cell cycle, 24 hours post-transfection cells were fixed using 66% ethanol (Decon Laboratories Inc., King of Prussia, PA) and stained with Propidium Iodide (PI) according to the manufacturer’s protocol (Thermo Fisher Scientific, F#10797). Stained cells were analyzed using BD LSRFortessa (BD Biosciences, Franklin Lakes, NJ), and cell cycle analysis was performed with ModFit LT4.1 (Verity Software House, Topsham, ME). A two-tailed Student’s t-test was performed using GraphPad Prism (Dotmatics). Figures were created using GraphPad Prism (Dotmatics).

## Results

### OCCC samples with concurrent endometriosis had unique molecular characteristics

While nearly half of all women with OCCC have endometriosis (22, 23), transcriptomic profiling studies have not examined OCCC samples from women with pathology-proven endometriosis. Only one transcriptomic study contained OCCC samples from women with a history of endometriosis (16), but the samples were not defined as pathology-proven nor were their transcriptomic profiles analyzed independently. Here, we focused on OCCC samples from women with pathologically-confirmed endometriosis. **Table 1** summarizes the clinical and pathological characteristics. OCCC with concurrent endometriosis was defined as having endometriosis at the time of staging surgery, listed on the pathology report. Women with OCCC were significantly older (median 53 years; range 39-79 years, *P<* 0.0001) than women with benign endometriomas (median 30.5 years; range 21-48 years). Women with OCCC and concurrent endometriosis did not differ in age (median 51 years; range 45-72 years, *P* = 0.15) from those without concurrent endometriosis (median 56.5 years; range 39-79). Using the Federation Internationale de Gynécologie et d’Obstétrique (FIGO) ovarian cancer staging system implemented in 2014 (72), there was no difference in the stage between the women with and without concurrent endometriosis (Fisher’s exact test = 1, *P* > 0.1). Thus, the OCCC samples were clinically similar except for concurrent endometriosis.

**Table 1:** Clinical characteristics of patient samples.

	Benign (*n* = 16)	Malignant (*n* = 19)		*P-*value
Median age, y (range)	30.5 (21-48)	53 (39-79)		^*^P< 0.0001
		With Endometriosis (*n* = 9)	Without Endometriosis (*n* = 10)	
Median age, y (range)		51 (45-72)	56.5 (39-79)	^*^P = 0.15
Stage				^*P* >0.10
I		4 (44%)	5 (50%)	
II		3 (33%)	2 (20%)	
III		2 (22%)	1 (10%)	
IV		0	2 (20%)	

^*^, Student’s t-test, one-sided, unpaired; ^Fisher’s exact test, comparing stage I to stage II+.

Poly-A bulk RNA and small RNA sequencing were performed on RNA isolated from specimens (*n* = 8), including endometrioma (*n* = 4) and OCCC with concurrent endometriosis (*n* = 4). Endometrioma samples were full cyst wall thickness samples taken from areas without gross pathology such as necrosis or abscess, with pathology-proven endometriosis without atypia and no evidence of ovarian cancer. OCCC samples were taken from adnexal masses, and samples were taken from areas free from gross endometriosis, necrosis, or abscess.

Poly-A bulk sequencing revealed over 437 million mapped reads (mean: 54,646,311 ± 6,117,236 mapped reads per clinical sample). There was no difference between endometrioma and OCCC with concurrent endometriosis samples regarding overall alignment rate, as all eight bulk RNA samples had greater than 97% of mapped reads aligned. To categorize mapped reads into RNA categories or feature counts, HISAT analysis was used (**Supplementary Table S4**). More reads were assigned in endometrioma (145,847,035 reads) than OCCC with concurrent endometriosis (124,361,541 reads, Student’s *t*-test, *P*< 0.01). OCCC with concurrent endometriosis had more reads assigned to mitochondrial RNA species (33,929,857 to 22,296,699; endometrioma, Student’s *t*-test, *P*< 0.01). More reads were assigned to protein-coding genes in benign endometrioma (98,430,773 reads) than OCCC with concurrent endometriosis (70,984,332 reads, Student’s *t*-test, *P*< 0.01).

**Figure 1:**
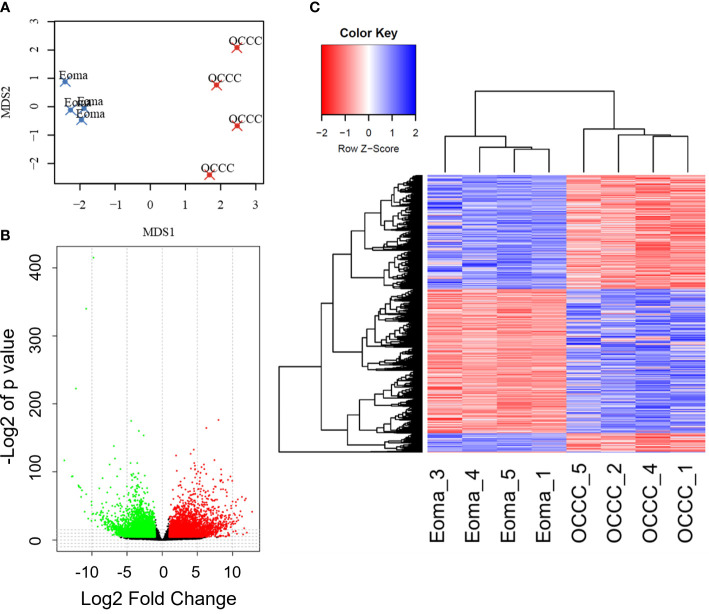
The OCCC with concurrent endometriosis transcriptomic profile is molecularly distinct from benign endometrioma. **(A)** Multidimensional scaling (MDS) plot of transcriptomic profiles for endometrioma (Eoma, blue X’s) and ovarian clear cell carcinoma (OCCC, red X’s) with concurrent endometriosis. **(B)** Volcano plot representation of protein-coding transcripts overexpressed (red dots), similarly expressed (black dots), and under-expressed (green dots) in OCCC with concurrent endometriosis versus endometriomas (*P_adj_* <0.05; Log2-Fold change<|1|. **(C)** Heat map representation of 5575 differentially expressed unique protein-coding gene transcripts overexpressed (red) and under-expressed (blue). Dendrogram of hierarchical clustering. Rows, protein-coding gene transcripts; columns, profiled samples.

Transcriptomic profiles of the endometrioma and OCCC with concurrent endometriosis samples were first evaluated using multidimensional scaling (MDS) analysis (73). The MDS plot shows a significant differential clustering of the OCCC samples with concurrent endometriosis from endometriomas (**Figure 1A**). This difference is most apparent at the MDS1 level. Similar clustering was noted on uniform manifold approximation and projection (UMAP) and principal component analysis (PCA) for dimension reduction plots (**Supplementary Figures S1A, B**). At a global level, malignant OCCC with concurrent endometriosis is molecularly distinct from benign endometriomas.

We directly compared transcriptomic profiles of OCCC with concurrent endometriosis (*n* = 4) to endometriomas (*n* = 4). Endometrioma was used as a comparison tissue due to its strong increase in risk for the development of OCCC, studies supporting increased molecular mutations in atypical endometriosis and concurrent OCCC, strong genomic correlation and causal relationship between endometriosis and OCCC, and the high incidence of concurrent endometriosis seen in women with OCCC (4, 11, 17, 18, 22, 23). Differential gene expression analysis was conducted with DESeq2. Significant differential expression was determined using a false discovery rate<0.05, giving 6865 protein-coding transcripts significantly differentially expressed. Limiting to log2 fold-change >|1| identified 2449 upregulated and 3131 downregulated unique protein-coding genes (**Figures 1B, C**, **Supplementary Tables S5, S6**). Hierarchical clustering shows that endometriomas cluster separately from OCCC with concurrent endometriosis (**Figure 1C**).

### Matrisome, secreted factors, cell cycle, and DNA replication pathways are dysregulated in OCCC with concurrent endometriosis

To explore potentially impactful molecular processes, we performed pathway enrichment analysis of the upregulated and downregulated genes using a hypergeometric test and Gene Set Enrichment Analysis (GSEA) against MSigDB v6 canonical pathway set, with a significant cutoff determined by *P*< 0.005 (46–48). Complete lists of the pathways significantly enriched by upregulated and downregulated genes are given in **Supplementary Tables S7**, **S8**. We observed a limited list of pathways from upregulated genes in OCCC with concurrent endometriosis (**Supplementary Table S7**). The upregulated genes showed significant enrichment in cell cycle and DNA replication pathways, including cyclin A B1-mediated G2-M transition, G1-S transcription, and E2F-mediated DNA replication (**Supplementary Figures S2A-C**). Previous work has shown that OCCC exhibited dysregulation of p27-related cell cycle effects (74). Important drivers of p27-related cell cycle dysregulation that were upregulated in OCCC with concurrent endometriosis include *SKP2* (S-phase kinase-associated protein 2, log2 fold-change = 1.3, *P* = 1.1e-2)*, CKS2* (CDC28 protein kinase regulatory subunit 2, log2 fold-change = 2.2, *P* = 6.6e-9)*, CCNA2* (Cyclin A2, log2 fold-change = 1.7, *P* = 2.9e-3), and *CCNE1* (Cyclin E1, log2 fold-change = 5.2, *P* = 8.5e-10). GSEA plots of the cyclin A-mediated G2-M transition (*P* = 8.95e-6) and E2F-mediated DNA replication (*P* = 8.61e-4) top enriched pathways from upregulated genes are shown in **Supplementary Figures S2B, C**. **Supplementary Table S9** lists the upregulated genes involved in the cell cycle with their fold change.

**Figure 2:**
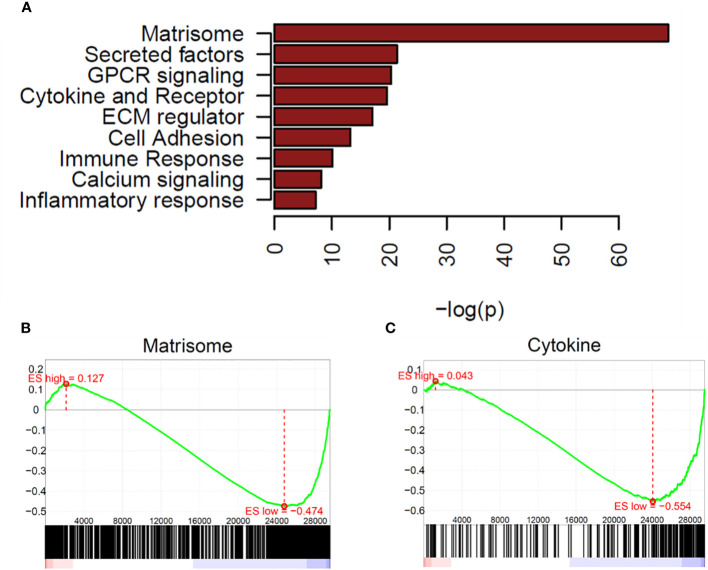
Matrisome and cytokine pathways are enriched in downregulated genes from OCCC with concurrent endometriosis. **(A)** Waterfall plot of significantly downregulated pathways in OCCC with concurrent endometriosis. Gene set enrichment plots for **(B)** NABA_MATRISOME and **(C)** KEGG_CYTOKINE_CYTOKINE_RECEPTOR_INTERACTION.

There were many more significantly downregulated pathways in OCCC with concurrent endometriosis. Significantly downregulated genes in OCCC with concurrent endometriosis showed significant enrichment in the pathways of matrisome, secreted factors, GPCR signaling, and cytokine-cytokine-receptor interaction (**Figure 2A**). GSEA plots of matrisome (*P* = 2.64e-69) and cytokine-cytokine receptor interaction (*P* = 2.43e-20) pathways from downregulated genes are shown in **Figures 2B, C**. Key genes involved in the cytokine-cytokine receptor interaction pathway are largely upregulated in endometriomas (7, 26, 75–78). **Supplementary Table S10** shows the downregulated genes in OCCC with concurrent endometriosis compared to endometrioma in the cytokine-cytokine receptor interaction pathway.

### Determining a model cell line of OCCC with concurrent endometriosis based on human gene expression by RNA sequencing

Genomically, OCCC cell lines frequently classify as OCCC rather than other histological subtypes of epithelial ovarian cancer (79–84). However, none of the widely shared or commercially available OCCC cell lines are characterized as being derived from OCCC with concurrent endometriosis. To determine which available cell lines most closely recapitulate our transcriptomic profiling data from OCCC with concurrent endometriosis, we used bioinformatic analysis of datasets from the Cancer Cell Line Encyclopedia (CCLE) (49). Gene expression datasets for ovarian carcinoma cell lines [endometrioid/clear-cell (IGROV-1, SKOV3, A2780), clear cell (TOV-21G, OVTOKO, OVISE, OVMANA, JHOC_5), p53-altered clear-cell (RMG-I, ES-2), endometrioid (TOV112D), high-grade serous (OVCAR8), and low-grade serous (HEYA8)], endometrial cancer cell lines (AN3CA, HEC1A, ISHIKAWA, HEC1B), and a leukemia cell line (JURKAT) were downloaded and used. For the RNA-seq gene expression data from the OCCC with concurrent endometriosis tissue samples and endometrioma samples, we utilized multiclass logistic regression with variable selection by L1 penalty. We selected pathways whose pathway activity scores (PAS) are most predictive of cancer types. PAS of the 394 cancer-types predictive pathways were computed for OCCC with concurrent endometriosis tissue, endometrioma, and the selected cell line samples. We computed a Pearson correlation between the PAS of 394 pathways in tissue and cell line data to measure the similarity.

**Figure 3:**
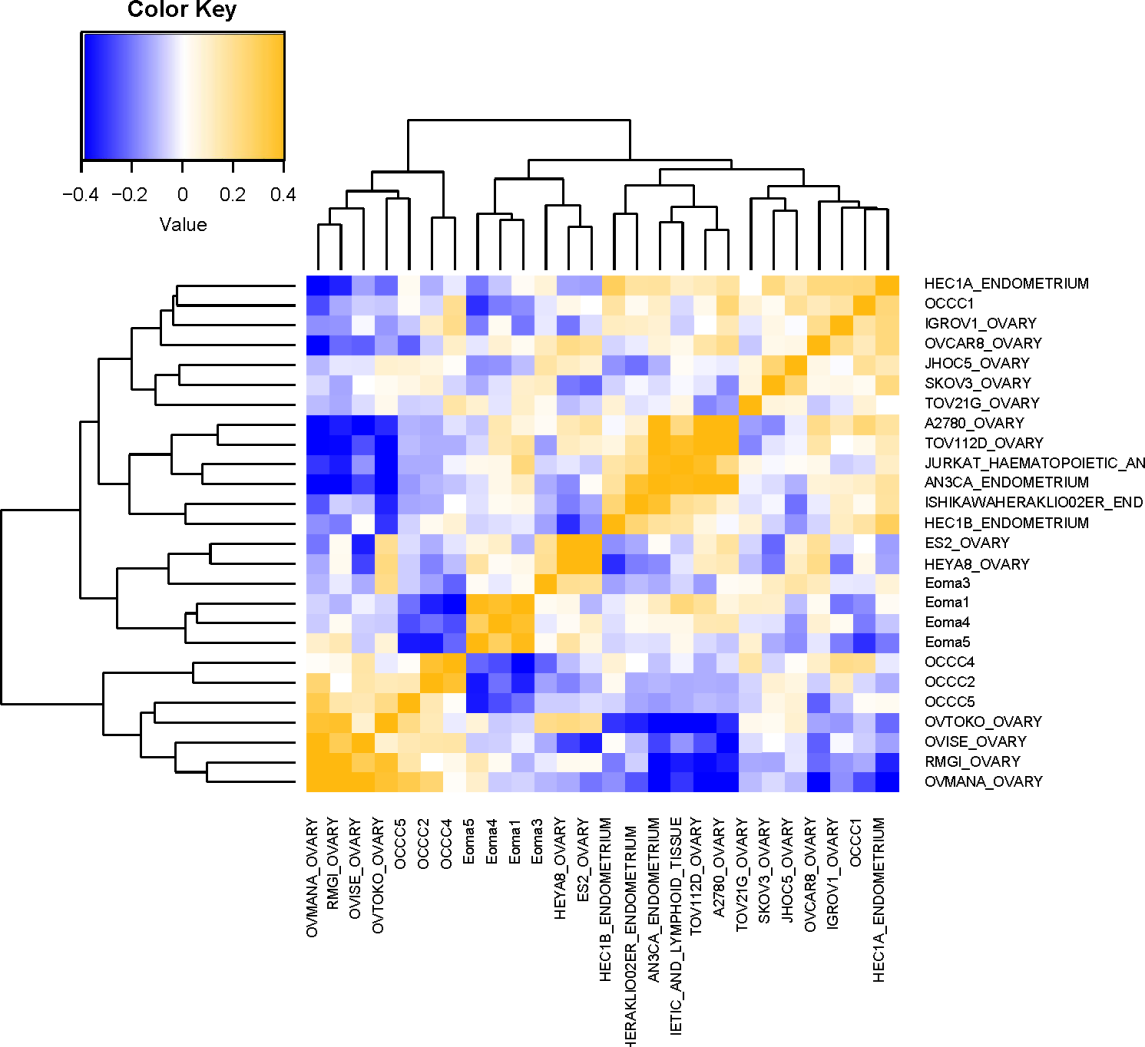
Molecular comparison of cell line transcriptomic profiles with clinical samples. Computational analysis of the molecular similarity of the clinical samples (Eoma, benign endometrioma or OCCC, OCCC with concurrent endometriosis) and cell lines from CCLE. A pathway activation score (PAS) was first computed for each pathway and each sample. Then the similarity between the samples was assessed by Pearson Correlation Coefficients (PCC) of the PAS. Cell line nomenclature is the CCLE name of the line_tissue type. A yellow color represents a higher PCC; blue, a lower PCC.

Examination of the PAS results (**Figure 3**) showed that endometrioma samples (Eoma1, Eoma4, Eoma5) clustered most closely to endometrioma sample 3 (Eoma3), ES2, and HEYA8. ES-2 is considered a p53-altered OCCC cell line (81, 85), and HEYA8 is considered a KRAS-mutant low-grade serous line (85). Studies suggest that both HEYA8 and ES-2 most closely represent low-grade serous (83). The dendrogram suggests that ES2 and HEYA8 cluster more closely to each other than Eoma3. OCCC samples with concurrent endometriosis (OCCC4, OCCC2) clustered together along with OCCC5, OVTOKO, OVISE, RMG-I, and OVMANA. OVTOKO, OVISE, and OVMANA are considered clear-cell ovarian cancer cell lines as they were derived from metastatic lesions of OCCC and contain a mutant ARID1A (57, 80, 83). RMG-I may be a p53-mutant clear cell type rather than a mutant ARID1A type (16, 83, 84). OCCC sample with concurrent endometriosis 1 (OCCC1) clustered with IGROV-1, JHOC5, SKOV3, and TOV-21G. IGROV-1 is derived from a mixed histology tumor and contains mutant ARID1A and PIK3CA and could be considered a clear cell-like line (81–83, 85). SKOV3 was derived from ascites of ovarian adenocarcinoma and contains mutant ARID1A and PIK3CA, and is frequently more closely associated with clear-cell tumors (63, 81–83, 85). TOV-21G is considered a clear cell line as it was derived from OCCC and contains mutations in ARID1A and PIK3CA (54, 81–83, 85) (**Supplementary Table S3**).

### OCCC with concurrent endometriosis samples have dysregulated miRNA expression

MiRNAs are impactful for their potential as disease biomarkers and role as upstream regulators of multiple signaling pathways in diseases of the female reproductive tract (24, 25). However, large-scale profiling of miRNAs has focused broadly on epithelial ovarian cancers without a direct analysis of OCCC, included only a small number of OCCC samples, or did not describe any samples with a history of or pathology-proven endometriosis (86–90). To complement our protein-coding transcriptomic studies, we profiled miRNAs on RNA isolated from clinical samples (*n* = 8), including endometrioma (*n* = 4) and OCCC with concurrent endometriosis (*n* = 4).

**Figure 4:**
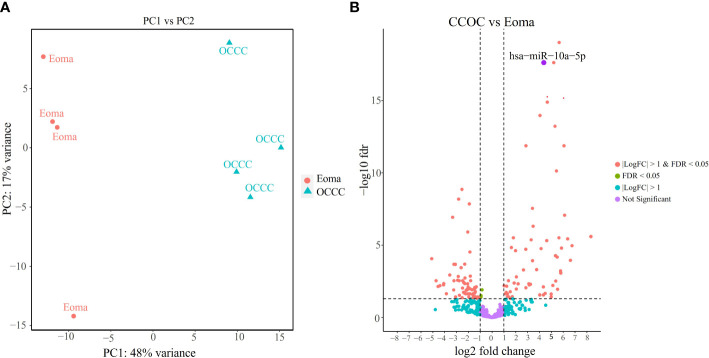
OCCC with concurrent endometriosis is molecularly distinct from benign endometrioma using miRNA expression. **(A)** Principal component (PC) analysis of malignant OCCC with concurrent endometriosis (triangles, OCCC) clusters separately from benign endometrioma samples (circles, Eoma), using PC1 and PC2. **(B)** Volcano plot of the significantly dysregulated miRNAs. Red represents miRNAs with *P*< 0.05, log2 fold-change > |1|. Blue dots represent miRNAs log2 fold-change > |1|. Green represents miRNAs with *P*< 0.05. Purple dots represent non-statistically significant miRNAs. The labeled dot represents hsa-miR-10a-5p.

Small RNA sequencing gave over 43 million reads (mean: 5,382,728 ± 644,063 mapped reads per clinical sample). There were no significant differences in the mirDeep2 total mapped count percentage between endometrioma and OCCC with concurrent endometriosis (**Supplementary Table S11**). Of the 2588 human mature miRNA molecules, 446 were expressed in at least one clinical sample. Principal component analysis (**Figure 4A**) with miRNA expression profiles showed PC1 and PC2 differential clustering of the OCCC with concurrent endometriosis from the benign endometrioma. Differential miRNA expression analysis was conducted with DESeq2. A comparison of dysregulated miRNAs between endometrioma and OCCC with endometriosis is shown on the volcano plot (**Figure 4B**). Significant differential expression was determined using a false discovery rate< 0.05, giving 128 significantly differentially expressed mature miRNA molecules. Limiting to log2 fold change > |1| identified 64 upregulated and 61 downregulated mature miRNA molecules (**Supplementary Tables S12**, **S13**).

Because fold change up- or downregulated is a relative number, we examined the most abundant miRNA molecules in endometriomas with statistically significant lower expression in OCCC with concurrent endometriosis. The most abundant miRNA in endometriomas was hsa-miR-143-3p, representing 14.5%. Hsa-miR-146b-5p represented nearly 5% (or 4.47%) of endometrioma miRNAs. **Table 2** shows the top ten downregulated mature miRNA molecules in OCCC with concurrent endometriosis. **Table 3** shows the top ten upregulated mature miRNA molecules in OCCC with concurrent endometriosis. MiR-10a-5p was the most abundant miRNA in OCCC with concurrent endometriosis, representing nearly a quarter of all miRNAs (21.5%). Other significantly abundant and upregulated miRNAs included hsa-miR-30a-5p (6.1%), two other miR-30 family members [hsa-miR30d-5p (0.71%) and hsa-miR-30c-5p (0.12%)], and hsa-miR-141-3p (1.35%). Three mature miRNA molecules had a log2 fold change >4 and were in the top ten in terms of abundance in OCCC with concurrent endometriosis: hsa-miR-10a-5p (log2 fold change = 4.37, *P* = 2.43e-18), hsa-miR-141-3p (log2 fold change = 4.67, *P* = 1.31e-15), and hsa-miR-183-5p (log2 fold change = 4.62, *P* = 4.90e-6).

**Table 2:** Top ten downregulated mature miRNAs in OCCC with concurrent endometriosis.

Mature miRNA	log2FoldChange	*P*_adj_
hsa-miR-143-3p	-2.81	6.61E-09
hsa-miR-127-3p	-1.91	1.41E-08
hsa-let-7c-5p	-3.09	2.11E-04
hsa-miR-99a-5p	-3.57	1.16E-03
hsa-miR-27b-3p	-1.80	2.87E-03
hsa-miR-199a-3p	-2.38	3.86E-03
hsa-miR-199b-3p	-2.38	3.86E-03
hsa-miR-146b-5p	-2.12	1.18E-02
hsa-miR-199a-5p	-2.18	1.31E-02
hsa-miR-23b-3p	-1.60	4.23E-02

**Table 3:** Top ten upregulated mature miRNA in OCCC with concurrent endometriosis.

Mature miRNA	log2FoldChange	*P*_adj_
hsa-miR-10a-5p	4.37	2.43E-18
hsa-miR-141-3p	4.67	1.31E-15
hsa-miR-30a-5p	2.87	1.37E-12
hsa-miR-183-5p	4.62	4.90E-06
hsa-miR-30d-5p	1.63	1.44E-05
hsa-miR-30c-5p	1.98	2.39E-05
hsa-miR-182-5p	3.77	4.94E-04
hsa-miR-98-5p	1.39	2.87E-03
hsa-miR-148b-3p	1.02	6.38E-03
hsa-miR-191-5p	1.24	6.87E-03

**Figure 5:**
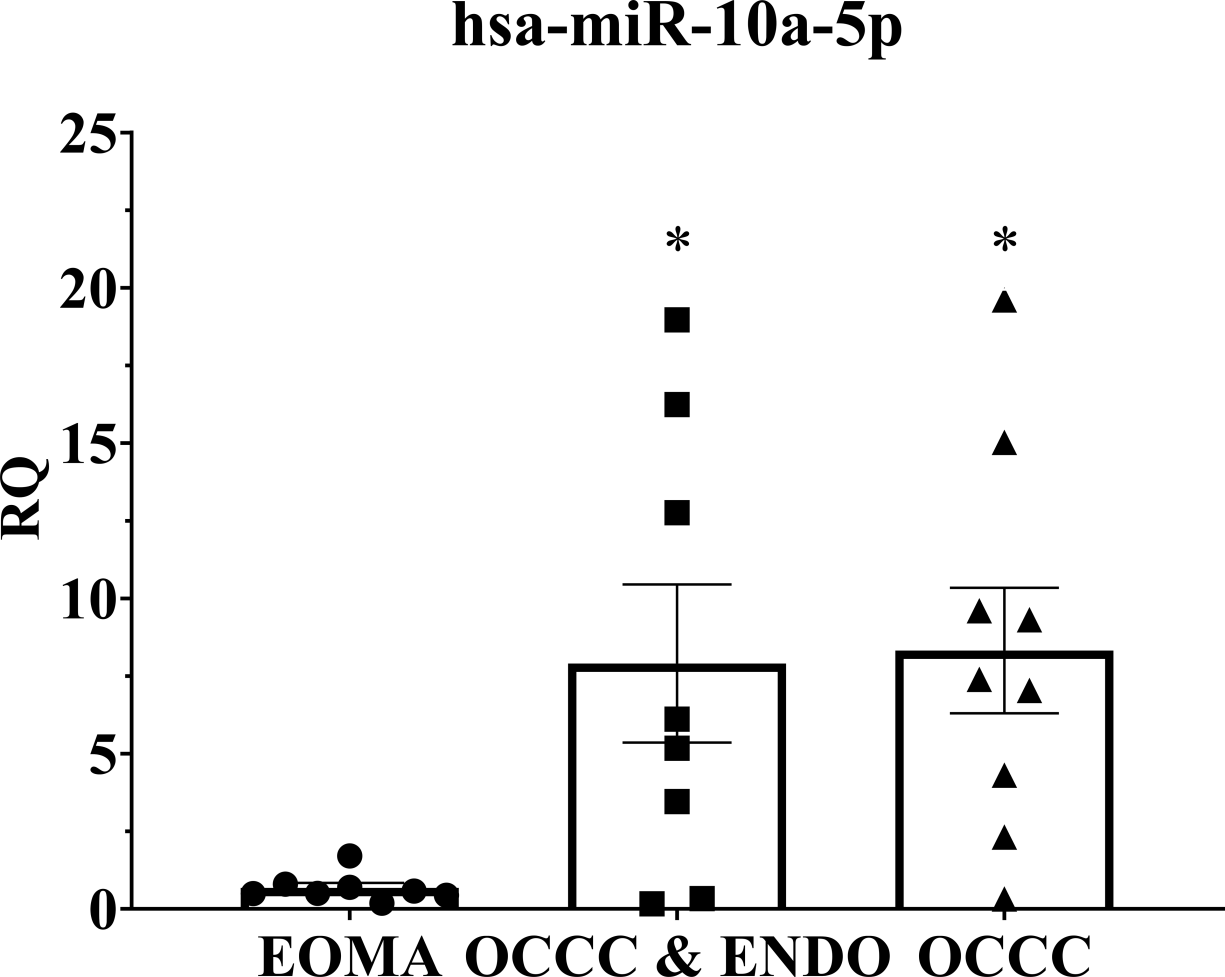
MiR-10a-5p is significantly upregulated in human tissue samples of ovarian clear cell carcinoma. Examination of miR-10a-5p expression in endometrioma (EOMA, *n* = 8), ovarian clear cell carcinoma from women with concurrent endometriosis (OCCC & ENDO, *n* = 8), and ovarian clear cell carcinoma from women without pathologically confirmed endometriosis (OCCC, *n* = 9). RQ, the relative quantity of hsa-miR-10a-5p to U6 snRNA, normalized to EOMA. Error bars represent ± SEM. *P< 0.05, one-way ANOVA.

Overexpression of miR-10a has been found in breast, cervical, acute myeloid leukemia, and pancreatic ductal adenocarcinomas (91–98). Further, miR-10a overexpression was correlated with an increased risk of recurrent breast cancer and decreased response to platinum agents *in vitro* (92, 96, 97, 99). Disease progression on first-line platinum therapy is a hallmark of OCCC, with response rates to chemotherapy ranging as low as 11% (100–105). While platinum resistance is the most common reason for death from recurrence across all ovarian cancers, progression on platinum therapy is more prevalent in OCCC (103, 104, 106). Thus, we explored the cellular and molecular effects of miR-10a in OCCC. QPCR expression showed that benign endometrioma exhibited a significantly lower expression of miR-10a-5p than OCCC with or without endometriosis (one-way ANOVA, *P*< 0.05, **Figure 5**). OCCC with concurrent endometriosis exhibited an 8-fold overexpression of miR-10a-5p compared to benign endometrioma (Student’s t-test, *P =* 0.01). However, there was no statistically significant difference in miR-10a-5p expression between OCCC with concurrent endometriosis and OCCC without endometriosis (Student’s t-test, *P* = 0.90).

### MiR-10a-5p expression in human OCCC cell lines correlates with carboplatin IC50

**Figure 6:**
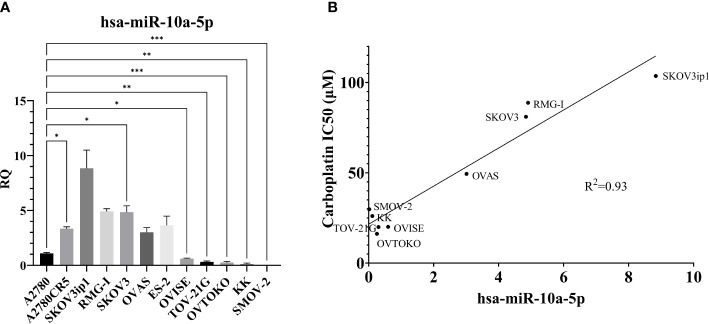
MiR-10a-5p expression across a panel of ovarian cancer cell lines. **(A)** RQ, the relative quantity of hsa-miR-10a-5p to U6 snRNA, normalized to A2780. ***P<0.001, **P<0.001, *P< 0.05, Brown-Forsythe and Welch ANOVA. A2780 (*n* = 6), A2780CR5 (*n* = 2), SKOV3ip1 (*n* = 3), RMG-I (*n* = 2), SKOV3 (*n* = 4), OVAS (*n* = 4), ES-2 (*n* = 4), OVISE (*n* = 9), TOV-21G (*n* = 4), OVTOKO (*n* = 7), KK (*n* = 3), and SMOV-2 (*n* = 6). **(B)** Correlation of miR-10a-5p expression to carboplatin IC50 in OCCC cell lines. RMG-I (*n* = 2), OVAS (*n* = 4), ES-2 OVISE (*n* = 9), TOV-21G (*n* = 2), OVTOKO (*n* = 7), KK (*n* = 2), and SMOV-2 (*n* = 6). Carboplatin IC50 is *n* ≥ 5 for each cell line.

To explore the role of miR-10a in OCCC, the expression of miR-10a-5p was examined in a panel of human ovarian cancer cell lines (**Figure 6A**). There appeared to be two groups of cell lines – those with low miR-10a expression (A2780, OVISE, TOV-21G, OVTOKO, KK, and SMOV-2) and those with high miR-10a expression (A2780CR5, SKOV3ip1, RMG-I, SKOV3, OVAS, ES-2). To confirm similar results, we analyzed the next-generation small RNA sequencing data from Nagaraja et al. (27). The relative expression of miR-10a in each cell line was similar to our expression. There was a low miR-10a expression in OVISE, TOV-21G, SMOV-2, and KK, and those with high relative miR-10a expression included RMG-I, ES-2, and OVAS (**Supplementary Figure S3**). ES-2 transcriptomically clustered with endometriomas (**Figure 3**) and exhibited relatively high expression of miR-10a in both our qPCR analyses (**Figure 6A**) and next-generation sequencing data from Nagaraja et al. (27). RMG-I and SKOV3 transcriptomically clustered with OCCC with concurrent endometriosis (**Figure 3**) and showed high expression of miR-10a (**Figure 6A**). On the other hand, OVISE, TOV-21G, and OVTOKO transcriptomically clustered with OCCC with concurrent endometriosis (**Figure 3**) but exhibited low expression of miR-10a (**Figure 6A**).

A2780CR5 cells are an isogenic line of A2780 that is resistant to platinum (62). Interestingly, the miR-10a-5p expression was 3.3-fold higher in the platinum-resistant line, A2780CR5 (Mann-Whitney, *P*< 0.01) than in A2780. Increased miR-10a expression has previously been correlated with platinum resistance in lung cancer (99, 107). As a result of this increased miR-10a-5p expression in the platinum-resistant line, carboplatin response was compared across OCCC cell lines. Carboplatin response was expressed as the half maximum inhibitory capacity (IC50) and correlated with miR-10a-5p expression. A positive correlation (R^2 = ^0.93) was found between miR-10a expression and carboplatin IC50 (**Figure 6B**).

### MiR-10a-5p overexpression decreases cellular proliferation.

SKOV3ip1cells are a xenograft-derived line of SKOV3. Previous work showed increased cellular proliferation of SKOV3ip1 cells compared to SKOV3 (56). The miR-10a-5p expression was almost 2-fold higher in SKOV3ip1 (un-paired t-test, *P*<0.05) than SKOV3. *In vitro* studies overexpressing miR-10a-5p showed potentially cancer type specific effects on cellular proliferation. For example, overexpression of miR-10a in melanoma, acute myeloid leukemia, and laryngeal squamous cell carcinoma cells decreased cellular proliferation in these cancers (108–110).

**Figure 7:**
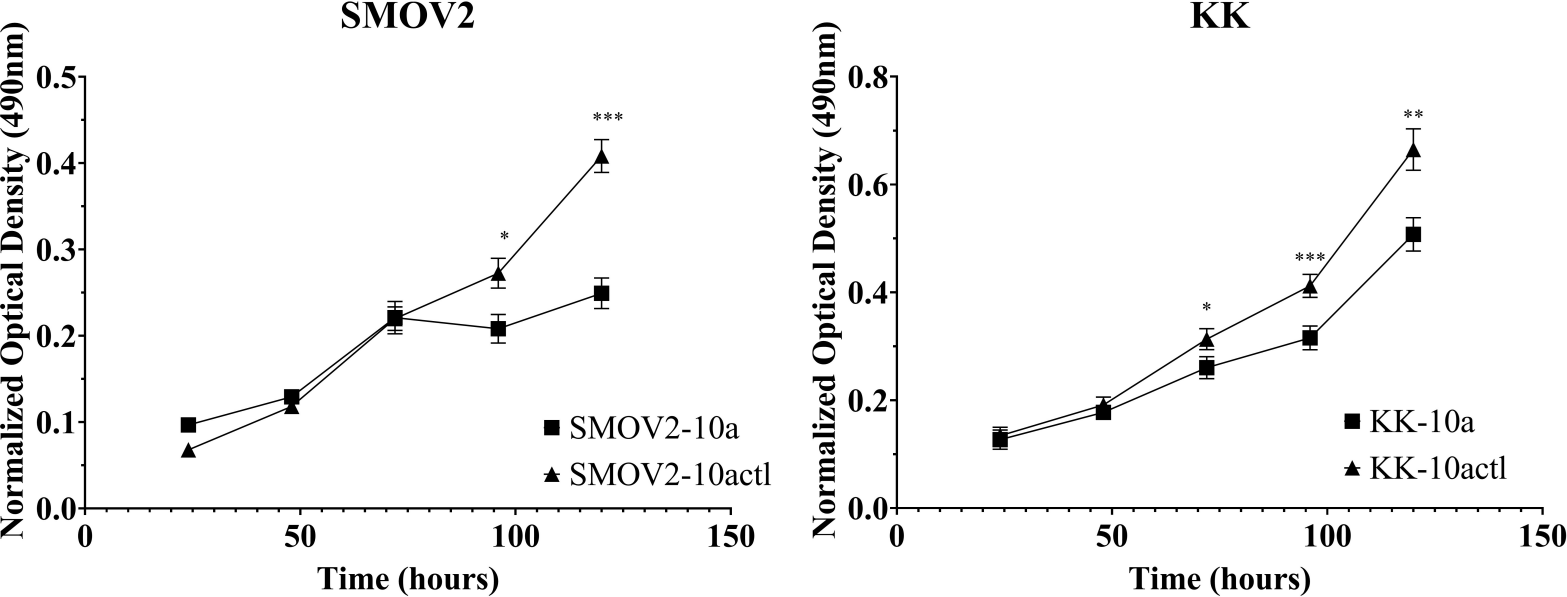
MiR-10a overexpression resulted in a significant decrease in cellular proliferation in SMOV2 and KK cells. MTS was normalized to the growth medium for each measurement. Cells transfected with mature miRNA mimic for miR-10a (SMOV2-10a and KK-10a) were compared to cells transfected with negative control #1 (SMOV2-10actl and KK-10actl) for each cell line and each time point. ***P< 0.0001, **P< 0.001, *P< 0.05, Student’s two-tailed t-test at each time point. *n* =6 for each cell line, timepoint, and transfection condition.

To study the effects of miR-10a overexpression on proliferation in OCCC cell lines, SMOV-2 and KK were transiently transfected with a mature miR-10a-5p mimic. After optimization of transfection conditions (data not shown), overexpression of miR-10a was confirmed (**Supplementary Figure S4**). SMOV-2 and KK miR-10a overexpressing cells (SMOV2-10a and KK-10a) had a statistically significant and sustained decrease in cellular proliferation compared to the non-targeting control transfected cells (SMOV2-10actl and KK-10actl) (**Figure 7**). For SMOV-2 cells, there was a statistically significant decrease in proliferation beginning at 96 hours. Overexpression of miR-10a in SMOV-2 cells showed an almost 2-fold increase in doubling time, from 42.8 hours to 84.9 hours. A statistically significant decrease in cell density was noted beginning at 72 hours in KK cells overexpressing miR-10a compared to a non-targeting control and continuing through 120 hours, there was a statistically significant decrease in proliferation. Overexpression of miR-10a in KK cells lengthened the double time from 40.9 hours to 47.2 hours.

### MiR-10a-5p overexpression shifts cells from S and G_2_ to G1 phase.

**Figure 8:**
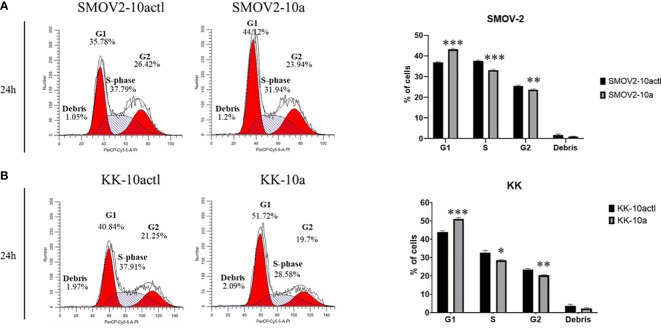
Overexpression of hsa-miR-10a-5p shifts cell cycle from S and G2 phase to G1. Cells transfected with mature miRNA mimic for miR-10a (SMOV2-10a and KK-10a) were compared to cells transfected with negative control #1 (SMOV2-10actl and KK-10actl) for each cell line. The flow histograms depict a representative biological replicate for **(A)** SMOV-2 and **(B)** KK. Graphical depictions represent *n* = 6 for each cell line and transfection condition. Statistical analysis was conducted using a 2-tailed Student’s *t-*test: ***P< 0.0001, **P< 0.001, *P< 0.05.

Cell cycle distribution was analyzed in SMOV-2 and KK cells overexpressing miR-10a-5p compared to non-targeting control transfected cells. SMOV-2 and KK cells overexpressing miR-10a-5p had a statistically significant increase in the G_1_ population and a decrease in S and G_2_ populations (*P*< 0.001) (**Figure 8**). SMOV2-10a had more than a 6% increase in the G_1_ population with a 4.5% decreased percent in S phase (*P<* 0.0001). KK-10a had a similar 7% increase in cells in the G_1_ phase, but KK-10a cells had a more distributed decrease in S phase (4%, *P* = 0.01) and G_2_ (3%, *P =* 0.002). The amount of cellular debris was not significantly changed in either SMOV-2 or KK samples (**Figure 8**).

### Predicted miR-10a-5p target genes dysregulated in OCCC with endometriosis play a role in signaling receptor binding.

MiRNA molecules are considered epigenetic regulators of gene expression (111). Overexpression of miRNA molecules leads to downregulation largely by destabilization of mRNA transcripts. Importantly, most mRNA molecules are targets of miRNAs (112). Each miRNA molecule has relative specificity of gene targets based on nucleotide sequence in the 3’UTR of the target gene. *In silico* prediction of genes that could be targeted by individual miRNA families is available in several databases. Target Scan predicts miRNA binding through complementary binding of the seed region of the mature miRNA molecule to the mRNA molecule, typically within the 3’UTR (66–68). As a slightly different algorithm for miRNA target gene predictions, miRDB uses *in silico* predicted miRNA binding to mRNA targets and downregulation of target gene expression from high-throughput sequencing data to identify putative targets. Additional predictions are added to miRDB from computational modeling and literature curation (69, 70). As another resource, miRTarBase uses natural language processing (NLP) to extract miRNA-predicted target gene data across the literature, to give miRNA-target interactions (MTIs). Examples of MTIs from miRTarBase include direct interaction studies of miRNA and target genes from CLIP-seq data, *in silico* seed sequence binding to mRNA from miRanda and miRBase, and experimental validation through reporter assays, western blots, or qPCR (71). To determine which dysregulated genes in OCCC with concurrent endometriosis were predicted targets of miR-10a-5p, miRNA:mRNA functional interaction prediction was undertaken using datasets from Target Scan v7.2, miRDB, and miRTarBase 2022 (65). Target Scan predicted 61 (**Supplementary Table S14**), miRDB predicted 62 (**Supplementary Table S15**), and miRTarBase predicted 67 (**Supplementary Table S16**) unique protein-coding genes downregulated in OCCC with concurrent endometriosis to be putative miR-10a-5p targets.

While 151 unique protein-coding genes were predicted to be miR-10a-5p target genes in at least one of the three algorithms, *BDNF* (brain-derived neurotrophic factor, log2 fold-change -2.42, *P* = 6.8e-5), *RORA* (RAR related orphan receptor A, log2 fold-change -1.98, *P* = 1.48e-7), *CSRNP3* (cysteine and serine-rich nuclear protein3, log2 fold-change -1.93, *P* = 1.57e-3), *CHL1* (cell adhesion molecule L1 like, log2 fold-change -3.08, *P* = 2.09e-5), *LIX1L* (limb and CNS expressed 1 like, log2 fold-change -1.13, *P* = 7.11e-5), and *RAP2A* (RAP2A, member of RAS oncogene family, log2 fold-change -1.52, *P* = 2.63e-5) were genes predicted to be miR-10a-5p targets in each of the three datasets. Using the 151 genes as input, the WEB-based Gene SeT AnaLysis Toolkit [WebGestalt (113)] revealed that the top network for miRNA targeting was the miR-10 family as expected (enrichment ratio = 13.67, **Supplementary Table S17**). Gene ontology molecular function analysis (**Supplementary Table S18**) showed enrichment in signaling receptor binding genes (enrichment ratio = 1.92, *P* = 1.5e-3). Pathway analysis (**Supplementary Table S18**) showed enrichment in cellular senescence genes (enrichment ratio = 4.71, *P* = 4.24e-3) and TGFβ signaling pathway (enrichment ratio = 7.17, *P* = 2.34e-3). A listing of the downregulated predicted miR-10a-5p target genes from the signaling receptor binding molecular function is listed in **Table 4**. Signaling receptor binding, cellular senescence, and TGFβ-signaling all involve the cell cycle.

**Table 4:** Putative miR-10a-5p target genes downregulated in OCCC with concurrent endometriosis within the receptor signaling pathway.

Gene Name	description	log2FoldChange	*P*_adj_
ACVR2A	activin A receptor type 2A	-1.52	5.14E-05
ARRDC3	arrestin domain containing 3	-2.11	1.38E-05
BAMBI	BMP and activin membrane bound inhibitor	-2.57	1.03E-10
BDNF	brain derived neurotrophic factor	-2.42	4.93E-04
DLG4	discs large MAGUK scaffold protein 4	-1.55	7.17E-06
EPHA4	EPH receptor A4	-3.29	2.75E-13
FEM1B	fem-1 homolog B	-1.56	3.87E-09
FHL2	four and a half LIM domains 2	-2.70	1.60E-08
FLRT2	fibronectin leucine rich transmembrane protein 2	-3.15	5.94E-13
GNAL	G protein subunit alpha L	-3.60	3.56E-06
HLA-E	major histocompatibility complex, class I, E	-1.32	4.39E-03
IL12A	interleukin 12A	-1.82	4.61E-02
IRS1	insulin receptor substrate 1	-1.54	1.10E-02
MMP14	matrix metallopeptidase 14	-2.98	9.40E-06
NEDD4	NEDD4 E3 ubiquitin protein ligase	-1.48	6.79E-05
PANX1	pannexin 1	-2.03	2.86E-06
PIK3CG	phosphatidylinositol-4,5-bisphosphate 3-kinase catalytic subunit gamma	-2.49	2.24E-05
PLSCR1	phospholipid scramblase 1	-1.37	4.91E-02
SERPINE1	serpin family E member 1	-4.78	1.10E-06
TGFB3	transforming growth factor beta 3	-3.11	2.05E-12
TNFRSF8	TNF receptor superfamily member 8	-2.93	4.28E-03
VDR	vitamin D receptor	-2.12	4.67E-02

### MiR-10a-5p overexpression downregulating genes involved in proliferation and cell cycle progression.

**Figure 9:**
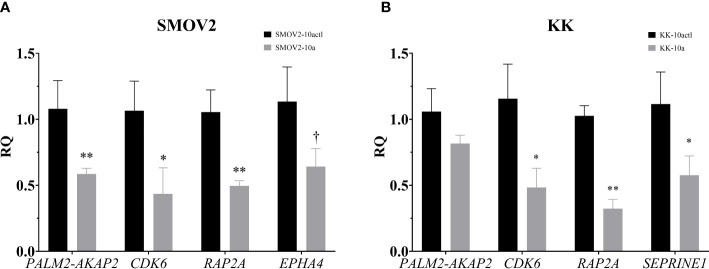
Putative miR-10a target genes, involved in cellular proliferation and the G1/S checkpoint, are downregulated with miR-10a-5p overexpression. Cells transfected with mature miRNA mimic for miR-10a (SMOV2-10a and KK-10a) were compared to cells transfected with negative control #1 (SMOV2-10actl and KK-10actl) for each cell line. **(A)** SMOV2 and **(B)** KK gene expression panels. RQ, the relative quantity of gene of interest to *ACTB*, normalized to negative control #1. Expression is plotted as mean ± SEM. Each gene was run with *n* = 6 for each cell line and treatment group. **P< 0.001, *P< 0.05, ^†^ *P*< 0.07 using unpaired Mann-Whitney or Welch’s one-tailed t-test within cell lines.

The 151 genes that were downregulated in OCCC with concurrent endometriosis and were putative miR-10a-5p target genes were hand-annotated for functional roles in proliferation or cell cycle. To examine the association of miR-10a-5p expression on these hand-selected putative target genes, mature miR-10a-5p was overexpressed in OCCC cell lines and target gene expression was examined by qPCR. Overexpression of miR-10a resulted in a nearly 2-fold decrease in *PALM2-AKAP2* in SMOV2 cells (*P<* 0.01, **Figure 9A**). A smaller, non-statistically significant effect was demonstrated in KK cells overexpressing miR-10a (*P* = 0.12, **Figure 9B**). *PALM2-AKAP2* is a newly named fusion gene with a yet unknown function, but it has been correlated with functions similar to the previously distinct *PALM2* and *AKAP2* genes, such as proliferation in colorectal cancer cell lines (114). Similarly, decreased *AKAP2* was shown to decrease cellular proliferation in ovarian cancer and decreased proliferation through regulation of *ERK1/2* (115, 116). Overexpression of miR-10a-5p was associated with a 2.4-fold decrease in Cyclin dependent kinase 6 (*CDK6*) gene expression in both SMOV2 and KK cells (*P<* 0.05, **Figures 9A, B**). CDK6 is a critical molecule for cellular proliferation and cell cycle progression from G1 to S phase (117, 118). Dysregulation of CDK6 is common in cancers and has been previously been implicated in dysfunctional proliferation and disease progression in ovarian carcinomas (119, 120). Overexpression of miR-10a-5p was associated with a 2-fold decrease in RAP2A, member of RAS oncogene family (*RAP2A*) gene expression in SMOV2 (*P<* 0.01, **Figure 9A**) and 3-fold decrease in KK cells (*P<* 0.001, **Figure 9B**). *RAP2A* is involved in cellular proliferation, has been positively correlated with increased platinum resistance in gastric cancer cells, and is a downstream target of *TP53* in cell cycle regulation (121–123). Overexpression of miR-10a-5p was associated with a more than 3-fold decrease in Serpin Family E Member 1 (*SERPINE1*) gene expression in KK cells (*P* = 0.01, **Figure 9B**). *SERPINE1* has been found to increase cancer cell proliferation through its regulation by miR-10a in clear cell renal carcinoma (124). Overexpression of miR-10a-5p was associated with a non-statistically significant decrease in Ephrin type A receptor 4 (*EPHA4*) gene expression in SMOV2 cells (*P*< 0.07, **Figure 9A**). *EPHA4* is a receptor involved in cancer cell proliferation in breast cancer cells through *AKT* signaling, where downregulation of *EPHA4* decreased proliferation and increasing *EPHA4* increased proliferation (125, 126). *EPHA4* is not expressed in KK cells (data not shown).

## Discussion

Ovarian carcinomas are the fifth leading cause of cancer-related death for women in the United States, accounting for over 13,000 deaths annually (127). While multi-platform analyses are attempting to categorize epithelial ovarian cancers beyond histology to discover molecular features that will modulate therapeutic benefit (1–3), current first-line therapy for women with ovarian carcinomas remains similar for all histological subtypes and includes surgical debulking to remove maximum tumor tissue and six cycles of carboplatin and paclitaxel or neoadjuvant chemotherapy (128). Fortunately, 70% of women with high-grade serous ovarian carcinomas show a complete response to these standard regimens (129). Unfortunately, up to 89% of women with OCCC show progression of disease with this standard protocol (100, 130, 131). These epidemiological data highlight a critical need for further understanding of the molecular features of OCCC to improve treatment options and discoveries.

Towards this need for understanding the molecular underpinnings of ovarian cancer, large sample size, multi-platform epigenetic (*i.e.*, DNA methylation, histone binding), genomic (*i.e.*, whole genome sequencing, exome sequencing, targeted gene sequencing, copy number variant), transcriptomic (*i.e.*, bulk RNA, small RNA, target gene expression), and proteomic (*i.e.*, targeted immunohistochemistry, reverse phase protein array) studies have been undertaken. Many of the multi-platform studies utilize the much more abundant sample numbers from high-grade serous tumors (3). Publicly-available transcriptomic datasets for OCCC are available within the Gene Expression Omnibus (**Supplementary Table S19**), and many of these datasets are published (16–21). Some studies on OCCC utilize transcriptomic profiles from OCCC cell lines (20, 27). For example, Yamaguchi et al. (20) created an OCCC signature from OCCC cell lines and compared it to multiple published or publicly available OCCC datasets. Nagaraja et al. (27) integrated transcriptomic microarray data with small RNA data from next-generation sequencing of a panel of OCCC cell lines compared to primary cultures of normal ovarian surface epithelium.

While up to 50% of OCCCs are associated with endometriosis (22, 23), most transcriptomic studies of primary tumors classified as OCCC do not characterize samples as coming from women with concurrent endometriosis, pathology-proven endometriosis or even a history of endometriosis (16–21). One of the studies that did delineate endometriosis was Bolton et al. (16) that performed the most extensive multi-platform sequencing of OCCC. They used both genomic (*n* = 421 samples) and transcriptomic (*n* = 211 samples) profiling. While more than 10% of their samples of OCCC were classified as coming from women with endometriosis (16), they did not analyze data from OCCC with concurrent endometriosis independently from those without endometriosis. This lack of concurrent endometriosis analysis could have been due to the endometriosis being based on patient-reported history and only confirmed by histology on samples from one institution. Shih et al. (18) included endometriosis from women without OCCC, endometriosis adjacent to OCCC, atypical endometriosis, and OCCC without endometriosis. The results showed that transcriptomic profiles from endometriosis adjacent to OCCC were most similar to atypical endometriosis. Similar to our results, they showed that endometriosis from women without OCCC was distinct from OCCC (18). Although they used laser capture microdissection, Shih et al. (18) did not examine OCCC samples from women with endometriosis. They focused more on the endometriosis samples and the transcriptomic transformation from endometriosis to atypical endometriosis to OCCC. Therefore, our study is unique for its inclusion and analyses of OCCC samples with pathologically confirmed endometriosis. Similar to Shih et al. (18), we used endometrioma samples without OCCC as a comparison tissue. All OCCC with concurrent endometriosis samples were primary tumor tissue with concurrent endometriosis confirmed by pathology reports with concurrent endometriosis. Unfortunately, these strict inclusion criteria and the limited availability of samples due to OCCC’s rarity restricted the sample size in our study. While we had OCCC samples without endometriosis available for study, we did not compare OCCC transcriptomic profiles with and without endometriosis. Previously published transcriptomic studies of OCCC without endometriosis (16–21) had a significant number of samples already profiled (>200) and publicly available within GEO. Thus, we focused on well-characterized samples of OCCC with pathology-proven concurrent endometriosis compared to ovarian endometriomas, as this analysis had not been undertaken previously. Zhang et al. (7) showed unique molecular profiling in ovarian endometrioid carcinomas with and without endometriosis. Future studies will focus on analyses of publicly available OCCC datasets from women with and without endometriosis. Additionally, future studies will focus on obtaining matched endometrioma and adjacent OCCC samples from the same patient. Given the likelihood of a contribution of the endometriotic tumor microenvironment, evaluation using spatial transcriptomics would provide considerable insight.

On our well-characterized samples, we performed poly-A bulk RNA. Like previous studies in ovarian endometrioid carcinomas with concurrent endometriosis (7), we identified signaling pathways dysregulated in OCCC with concurrent endometriosis, including cytokine-cytokine receptor interaction, GPCR signaling, matrisome, and cell cycle and DNA repair pathways. Ovarian cancer cell lines are widely used as a model for epithelial ovarian carcinomas, and multiple studies have shown representative mutational, transcriptomic, and histological similarities between primary OCCC and OCCC cell lines (54, 57, 79–85). Many of these cell lines, including those derived from endometriosis-associated ovarian carcinomas (OCCC and ovarian endometrioid carcinomas), are not characterized by endometriosis status. In order to identify cell lines as the closest model of OCCC with concurrent endometriosis, our study utilized publicly available transcriptomic data for cancer cell lines, differentially expressed genes, and pathway activation scores. From this analysis we found that the OVTOKO, OVISE, RMG1, OVMANA, TOV21G, IGROV1, and JHOC5 cell lines are the cell lines with the most similar molecular profile to our OCCC with concurrent endometriosis dataset. Future studies with larger sample sizes of clinical samples would allow for a more comprehensive study of the subtle molecular nuances of the cell lines.

As a multi-platform transcriptomic approach, we also profiled small RNA molecules. To our knowledge, this represents the first small RNA sequencing from OCCC with concurrent endometriosis. Small RNA sequencing identified miR-141-3p, miR-183-5p, and miR-10a-5p as the top three most upregulated miRNAs in OCCC with concurrent endometriosis. MiR-141-3p overexpression has been demonstrated in a panel of platinum-resistant cell ovarian cancer lines (132). Further, increased expression of miR-141-3p was associated with increased cellular proliferation in esophageal cancer (133). Like miR-141-3p, studies of miR-183-5p in ovarian carcinoma are limited, but bioinformatic analysis in high-grade serous ovarian carcinoma correlated miR-183-5p with platinum-resistance (134). The specific role of these miRNAs in OCCC is currently unknown and will be crucial components in future studies.

**Figure 10:**
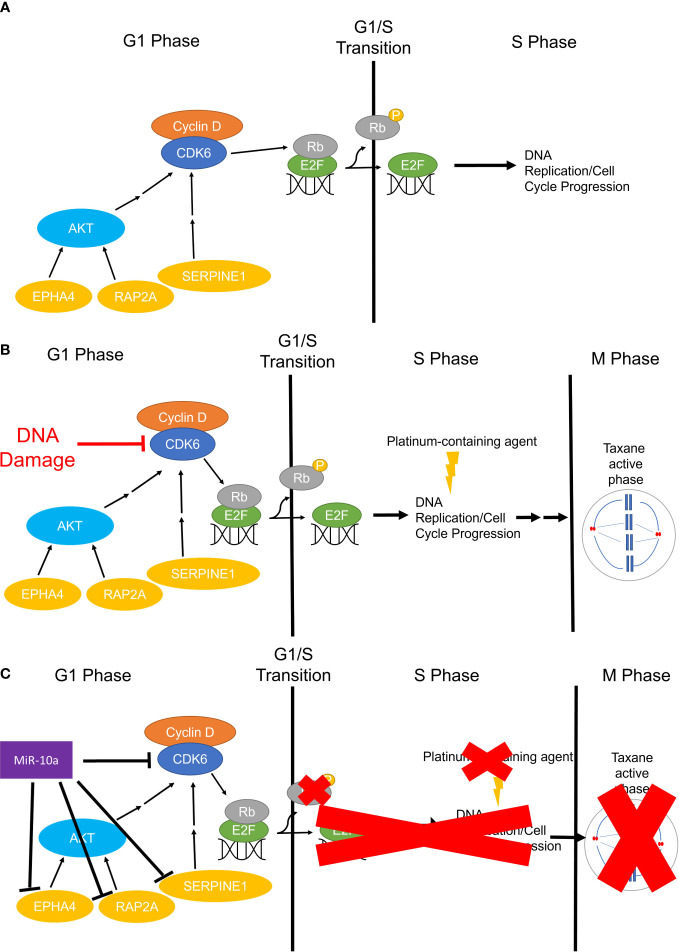
The working hypothesis for the mechanism of miR-10a-5p overexpression in OCCC G_0_ or G_1_/S Checkpoint. **(A)** Simplified representation of cell cycle progression function in non-cancerous cells, whereby CDK6 phosphorylates Rb freeing E2F for DNA replication in the S phase. **(B)** Infographic representation of cell cycle progression in non-malignant cells treated with platinum and/or taxane-containing agents. Cells will sustain DNA and/or microtubule damage resulting in no continued progression through the cell cycle and subsequent cell death. **(C)** Represents the working hypothesis for cell cycle progression in miR-10a-5p overexpressing OCCC cells. MiR-10a-5p downregulates *CDK6* and other important regulators of the cell cycle slowing or halting phosphorylation of Rb leading to inactive or prolonged inactivation of E2F and transition to S Phase and DNA replication. Cells slowed in G_1_ or senescing in G_0_ miss the critical chemotherapeutic effects in the S and M phases (red “X”s).

MiR-10a-5p was the most abundant miRNA in OCCC with concurrent endometriosis, comprising 21% of the miRNA molecules. MiR-10a has been found to be upregulated in primary ductal breast carcinomas, squamous cell cervical carcinomas, acute myeloid leukemia, and pancreatic ductal adenocarcinomas and correlated with disease progression and platinum-resistance (91–98, 135, 136). Similar to other cancers, our study observed a strong, positive relationship between miR-10a-5p expression and platinum response in a panel of OCCC cell lines (R^2 = ^0.93). Focusing on benign disease and ovarian function, previous studies have shown that miR-10a-5p expression is significantly lower in endometriomas compared to matched and unmatched eutopic endometrium (26, 137). Further, increased expression of miR-10a-5p in granulosa cells resulted in decreased proliferation (138), consistent with our results in OCCC cell lines. Moreover, increased expression of miR-10a-5p in granulosa cells led to cell cycle deficiencies, mediated through indirect regulation of cyclin-dependent kinase 2 (138). Dysregulated genes in the cell cycle and DNA repair pathways have been implicated in OCCC and associated with its disease progression and platinum-resistant phenotype: *RAP2A* (139–141), *CDK6* (119, 120, 142)*, SERPINE1* (143, 144), and *EPHA4* (145–148) are involved in disease progression, drug response, and markers of progression. Overexpression of miR-10a-5p in OCCC cell lines showed an associated decrease in the expression of these putative miR-10a-5p target genes. These studies do not prove a direct effect of miR-10a-5p on this target gene expression. However, several studies have shown direct effects or associated effects of miR-10a on *SERPINE1* (124) and *EPHA4* (148, 149) gene expression. **Figure 10** shows our working hypothesis of the role of miR-10a-5p effects on cell cycle progression in OCCC. In summary, we found a significant decrease in cellular proliferation with overexpression of miR-10a-5p. This decrease in proliferation may be due to a deficit in the G1/S checkpoint as a significant increase in cell population in G_1_ was seen in cell cycle analysis while also having a significant decrease in cells in S and G_2_ phases. Upon further evaluation of miR-10a target genes involved in proliferation, genes involved in regulating the G_1_/S checkpoint were downregulated in SMOV-2 and KK cells transfected to overexpress miR-10a. More specifically, the miR-10a-5p target gene, *CDK6*, is well known for its regulation of a cell’s progression to the S phase through its dimer with Cyclin-dependent kinase 4 (*CDK4*) (150). Other genes, including *EPHA4* (126), *RAP2A* (123), and *SERPINE1* (151) have been implicated in cell cycle progression. Of particular interest to the increased number of cells in G_1_ are *CDK6* and *EPHA4* with downregulation of both being linked to cells remaining in G_0_/G_1_ (126, 152). The increase in this population of cells is clinically significant for the phases of the cell cycle in which platinum and taxanes show efficacy (153, 154). These drugs are commonly effective during S and M phases causing DNA and mitotic spindle damage leading to cell death. However, if OCCC cells are overexpressing miR-10a and stuck in a senescent or earlier stage of the cell cycle for an extended period, they will be less likely to undergo damage and death from cytotoxic platinum and taxane agents, leading to resistant tumors. Future studies, including the evaluation of phosphorylated proteins, are needed to explore this hypothesis further.

## Data availability statement

The data presented in the study are deposited in the Gene Expression Omnibus (GEO), accession number GSE230956.

## Ethics statement

The studies involving human participants were reviewed and approved by Institutional Review Board (IRB) at Indiana University (#1812764043). Written informed consent for participation was not required for this study in accordance with the national legislation and the institutional requirements.

## Author contributions

KC, XW, AB, DR, CZ, and SH contributed to conception and design of the study. AB performed RNA sequencing analysis. KC, XW, YK, ND, MM, and SH performed bench experiments. KC, AB, DR, CZ, KS, CC, and SH performed bioinformatic and computational analysis. KC and SH wrote the first draft of the manuscript. KC, XW, YK, ND, MM, CZ, KS, AB, DR, and SH wrote sections of the manuscript. All authors contributed to the article and approved the submitted version.
