# Supplementary material for: Transcriptomic analyses of ovarian clear-cell carcinoma with concurrent endometriosis
Source: Front Endocrinol (Lausanne). 2023 Aug 9;14:1162786. doi: 10.3389/fendo.2023.1162786 (PMC10445169; doi:10.3389/fendo.2023.1162786)
Supplement: Supplementary file 3 [file DataSheet_3.docx]

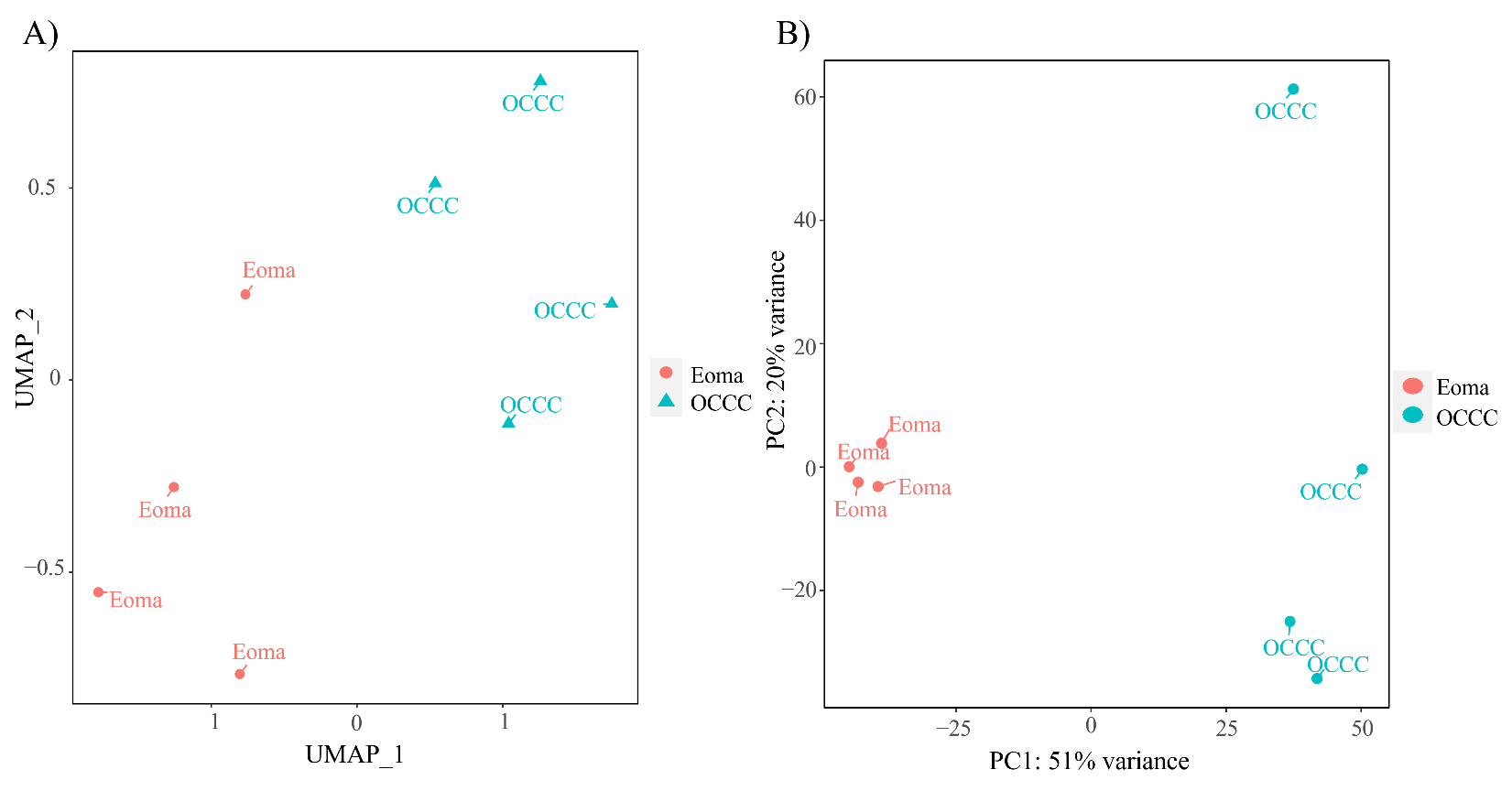


**Supplementary Figure S1. OCCC with concurrent endometriosis differentially clusters from benign endometrioma. A)** Uniform manifold approximation and projection (UMAP) plot of malignant OCCC with concurrent endometriosis (blue triangles) clusters separately from benign endometrioma (red circles) using UMAP1 and UMAP2. **B)** Similarly, principal component (PC) analysis of malignant OCCC with concurrent endometriosis (blue circles) clusters separately from benign endometrioma samples (red circles), using PC1 and PC2.


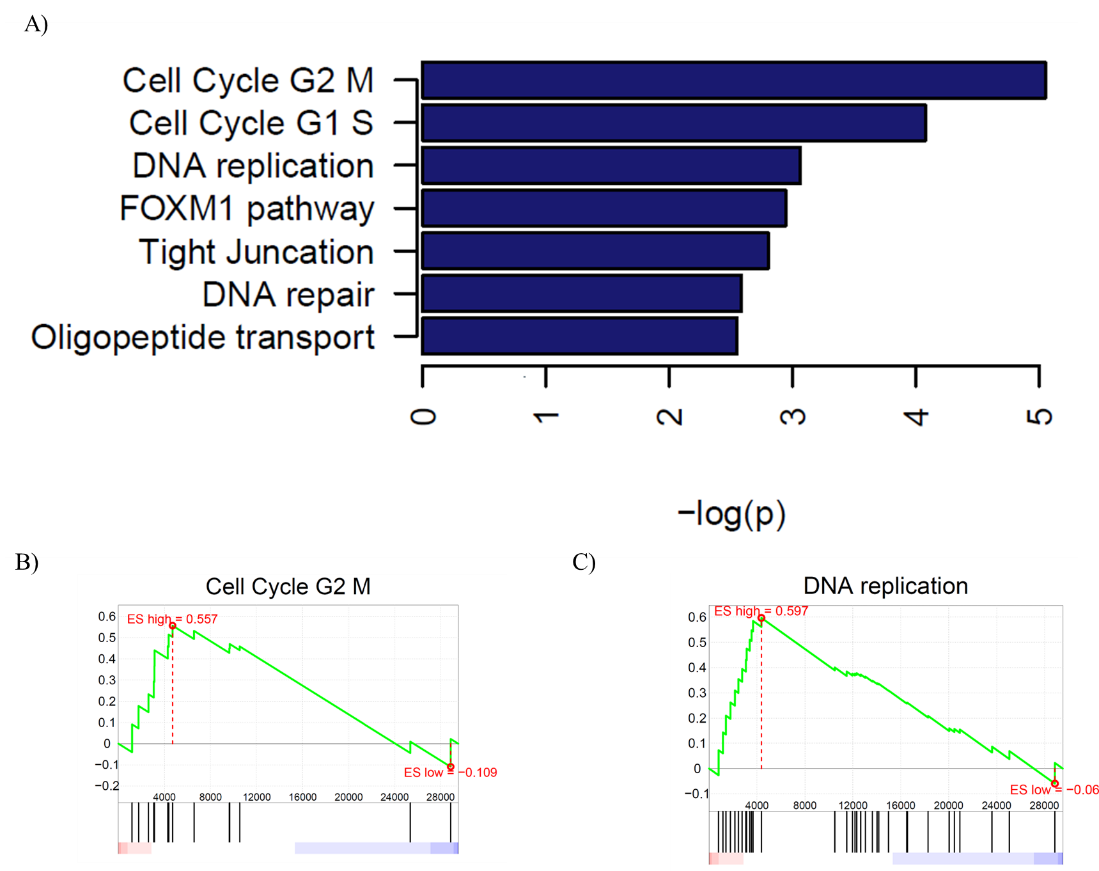


**Supplementary Figure 2. Cell cycle and DNA replication/repair pathways are enriched in upregulated genes from OCCC with concurrent endometriosis**. **A)** Waterfall plot of significantly upregulated pathways in OCCC with concurrent endometriosis. Gene set enrichment plots for **B)** REACTOME_CYCLIN_A_B1_ASSOCIATED_EVENTS_DURING_G2_M_TRANSITION and **C)** REACTOME_E2F_MEDIATED_REGULATION_OF_DNA_REPLICATION.

**Supplementary Figure S3. Expression of hsa-miR-10a-5p across OCCC cell lines.** A plot of read counts from Nagaraja *et al*., 2010. RQ, the relative quantity of hsa-miR-10a-5p normalized read counts in each cell line normalized to primary cultures of normal ovarian surface epithelium (NOSE). Error bars represent ± SEM. Each cell line *n* = 1; NOSE, *n* = 4.


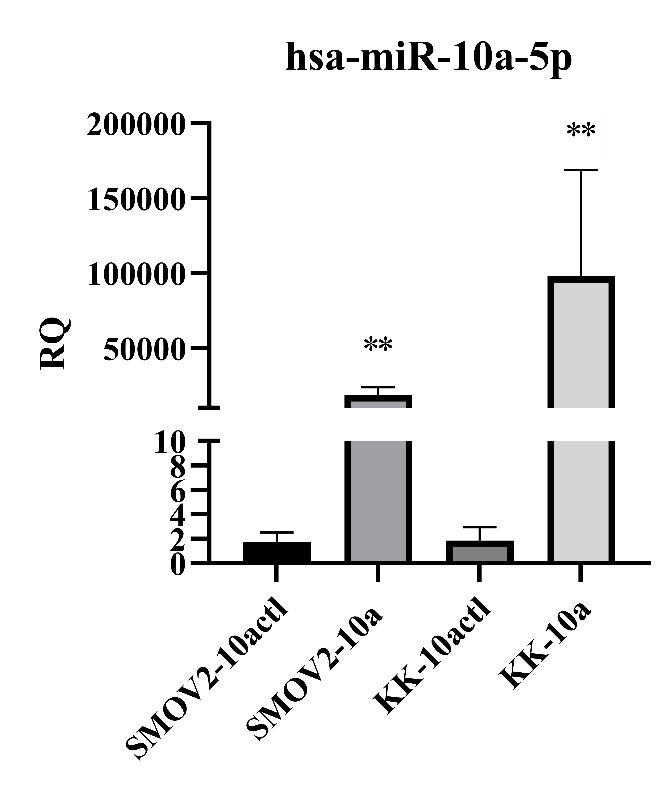


**Supplementary Figure S4. MiR-10a-5p is significantly overexpressed in SMOV-2 and KK cells transfected with a miR-10a-5p mimic.** RQ, the relative quantity of hsa-miR-10a-5p to U6 snRNA, normalized to each negative control transfected line. Cells transfected with mature miRNA mimics for miR-10a (SMOV2-10a and KK-10a) were compared to cells transfected with negative control #1 (SMOV2-10actl and KK-10actl). Error bars represent ± SEM. KK transfection, *n*=6; SMOV2-10a, *n*=5. **p<0.05, using Mann-Whitney two-tailed test.
